# From “Step Away” to “Stand Down”: Tailoring a Smartphone App for Self-Management of Hazardous Drinking for Veterans

**DOI:** 10.2196/16062

**Published:** 2020-02-13

**Authors:** Daniel Blonigen, Brooke Harris-Olenak, Eric Kuhn, Keith Humphreys, Christine Timko, Patrick Dulin

**Affiliations:** 1 Department of Veterans Affairs Palo Alto Health Care System Menlo Park, CA United States; 2 University of Alaska-Anchorage Anchorage, AK United States

**Keywords:** veterans, hazardous drinking, Step Away, Stand Down, peer support

## Abstract

**Background:**

US military veterans who screen positive for hazardous drinking during primary care visits may benefit from a mobile app. Step Away is an evidence-based mobile intervention system for the self-management of hazardous drinking. However, Step Away was not designed for veterans, and differences between veterans and civilians could limit the reach and effectiveness of the app with this population.

**Objective:**

The primary objective of this study was to repurpose Step Away to address the needs and preferences of the veteran primary care population. The Method for Program Adaptation through Community Engagement (M-PACE) model was used to guide the adaptation process. This model can serve as a generalizable approach that other researchers and intervention developers can follow to systematically tailor mobile health tools for a new population.

**Methods:**

Veteran patients who screened positive for hazardous drinking during a primary care visit (n=12) and peer providers employed by the US Veterans Health Administration (n=11) were recruited to systematically review Step Away and provide feedback on its content and presentation via Web-based surveys and a semistructured interview. Participant feedback was reviewed through an iterative process by key stakeholders who adjudicated which suggested modifications to the app could enhance engagement and effectiveness with veterans while maintaining program integrity.

**Results:**

Usability ratings of the individual modules of Step Away were uniformly positive across patients and peers, as was the perceived utility of the app overall. Personalized feedback on the health consequences and costs of drinking, options for customization, and the measurement-based care capabilities of the app were viewed as facilitators of engagement. Conversely, lengthy text, small font, and a lack of interactive features were viewed as potential barriers with the older primary care population. Modifications to create a veteran version of the app (*Stand Down: Think Before You Drink*) included altering the appearance of the app to incorporate more veteran-centric content, adding links and options for resources and activities for veterans, and reducing the amount of text and adding veteran-specific references and common concerns and triggers for drinking in this population.

**Conclusions:**

The M-PACE model provided a systematic approach to repurpose Step Away to fit the needs and preferences of veteran primary care patients who engage in hazardous drinking. Stand Down may serve as an innovative, low-cost means of expanding access to care for veterans who engage in hazardous drinking.

## Introduction

### Overview

The evidence for various smartphone apps to improve users’ mental health is expanding [1,2]. Nonetheless, for many mobile apps, the patient population for which they were developed and validated differs in important ways from subpopulations that may also benefit from these apps. To cite one of many possible examples, an app developed on a young adult sample would not necessarily be as appealing to the elderly population, thus creating barriers to implementation [3]. Consequently, tailoring mobile apps to maximize reach and effectiveness with new populations defined by demographic and life experience factors is essential. In this paper, we outline a systematic approach to tailoring a mobile app for populations for which it was not originally designed [4]. To illustrate this approach, we describe a mobile-based intervention system for self-management of drinking problems–*Step Away* [5-8]–that was originally developed for the general population. We outline our efforts to repurpose Step Away’s content and presentation to fit the characteristics, needs, and preferences of US veterans to maximize engagement and effectiveness with this population.

### Hazardous Drinking Among US Veterans: Using Mobile Apps to Improve Access to Care

Hazardous drinking*—*a pattern of alcohol use that increases an individual’s risk for negative health consequences—is a significant public health problem and financial burden on health care systems and the society at large [9]. Hazardous drinking is commonly assessed using the Alcohol Use Disorder Identification Test for Consumption (AUDIT-C) [10], which is also an effective screening questionnaire for identifying those with a past-year diagnosis of an alcohol use disorder (AUD) [11]. In the US Veterans Health Administration (VHA), 15% to 30% of veterans screen positive for hazardous drinking on the AUDIT-C during a primary care visit; however, most of these patients do not obtain treatment for their drinking despite wide availability of these services in VHA [12].

Mobile apps offer an innovative and low cost means of expanding access to care for veterans who engage in hazardous drinking. Such apps have the benefit of overcoming key barriers to care access. For example, an app would make appointments unnecessary, thus reducing the burden on both the VHA (eg, staffing) and patients (eg, travel costs) [13,14]. Providing veterans with an option for care that is private, self-directed, and available as needed can also address the stigma that many patients have in regard to alcohol use treatment [15]. There is a need for an evidence-based mobile app that is customized and targeted toward veterans to help them reduce or abstain from alcohol use.

### Step Away: Mobile App for Hazardous Drinking

Step Away is a mobile-based intervention program for individuals who want to reduce or abstain from drinking but are unable or unwilling to receive in-person care [5,16]. Development of Step Away was guided by the empirically supported alcohol interventions of motivational enhancement and cognitive-behavioral therapies, particularly relapse prevention and community reinforcement approaches [17,18]. For example, the app provides a comprehensive assessment of drinking patterns, personalized feedback to enhance awareness of drinking-related problems, tracking progress toward drinking goals, and in-the-moment tools to help manage cravings and triggers. Step Away is applicable to individuals with a range of alcohol use severity as it allows a choice of either moderation or abstinence as a drinking goal.

Published research on Step Away has focused exclusively on the prototype version. In a pilot randomized controlled trial of 54 adults with an AUD diagnosis, the use of the prototype version was associated with significant reductions in heavy drinking days and drinks per drinking day over 6 weeks and more percentage days abstinent relative to use of a Web-based program (Drinker’s Check-up) plus bibliotherapy [7]. On the basis of the usability ratings and system usage data, the prototype was subsequently modified, named “Step Away,” and is now available for download on iPhones and other iOS (iPhone Operating System) platform mobile devices [19]. Step Away contains 10 modules: (1) drinking patterns, (2) goals, (3) rewards, (4) cravings, (5) strategies, (6) support persons, (7) reminders, (8) high risk, (9) moods, and (10) new activities. Step Away also includes a *Get Help* feature that provides strategies and resources for individuals who need immediate assistance to manage cravings or other problems, and Daily and Weekly Interviews to help individuals monitor their alcohol use and track progress toward their drinking goal [16]. The acceptability and usability of the current version of Step Away has not been reported, though recent unpublished work from New Zealand and the United States indicates that acceptability of this version is high (P Dulin, personal communication).

### Tailoring Step Away for US Veterans

Step Away was not designed for US veterans *per se.* Differences between veterans and civilians highlight the value of repurposing Step Away to address the characteristics, needs, and preferences of veterans. For example, modifications to the appearance of mobile health (mHealth) tools to include references to military culture can increase the likelihood that a veteran will identify with the program’s content and engage with the tool [20]. Differences in the demographics and mental health needs of veterans (vs civilians) may also be relevant. To date, the development and validation of Step Away has been limited to adults aged 18 to 45 years, whereas the majority of veterans who screen positive for hazardous drinking during a primary care visit are in mid-to-late life [21,22]. Consequently, less text or larger font may be needed to facilitate usability among the older veteran population [3]. Relative to young adults, older adults who engage in hazardous drinking also struggle with more comorbid medical conditions [23] and may therefore benefit from feedback on the health consequences of drinking. Finally, the rate of trauma exposure and the comorbidity between hazardous drinking and posttraumatic stress disorder is higher among veterans than in the general population [24]. Therefore, references to the association between trauma and alcohol use may enhance the relevance of the app for some veterans.

### This Study

The objective of this study was to repurpose the Step Away mobile intervention system to create a version of the app that would maximize engagement and effectiveness with US veterans. To achieve this objective, we applied the Method for Program Adaptation through Community Engagement (M-PACE) model [4]. M-PACE is a systematic approach to soliciting comprehensive feedback from stakeholders on potential changes to an intervention to address differences in a target population and deciding on which changes to implement. Per the M-PACE model, incorporating consumer feedback into the adaptation of an intervention, along with feedback from relevant experts and program facilitators, results in improved acceptability and utility of the intervention while maintaining program fidelity. The goal is to enhance engagement of the intervention in a new population without diluting the intervention’s effectiveness.

The long-term goal of this research program is to integrate the use of a veteran version of Step Away with telephone support from peer providers. Such providers would be veterans who are currently in recovery from addiction and have been trained to serve other veterans who are actively struggling with these issues. By encouraging utilization of the app and monitoring Veterans’ progress toward their recovery goals, a peer provider can provide supportive accountability to patients who use Step Away, thus increasing the reach and sustainability of this app with the veteran population [25]. Therefore, the samples for this study were veteran patients who screened positive for hazardous drinking during a VHA primary care visit and peer providers who serve veterans with addiction.

## Methods

### Participants

#### Patients

VHA administrative data were used to identify eligible patients who received a positive screen on the AUDIT-C (scores of >4 for women and >5 for men) during a visit to a VHA primary care clinic between July and October 2017. A total of 150 eligible patients were sent a study invitation letter in the mail; patients who did not respond to the letter were called approximately 1 week later. Overall, 81 patients (81/150, 54.0%) opted out, 33 (33/150, 22.0%) did not respond to a voicemail message, and 13 (13/150, 8.7%) were unable to be reached because of their message inbox being full or the phone number listed in patients’ medical records being incorrect or not in service. Furthermore, 23 patients (23/150, 15.3%) were able to be reached and expressed interest in the study. Among this pool of interested patients, quota sampling based on gender, age, and race and ethnicity was used to select a representative sample of VHA primary care patients who screened positive for hazardous drinking. A total of 13 patients were initially enrolled, and 1 withdrew, yielding a sample of 12 (see sample characteristics in Table 1). Patient participants were mostly male (11/12, 92%), white (non-Hispanic; 7/12, 58%), college educated (9/12, 75%), and 58.9 years old, on average (SD 19.4). At the time of enrollment, 4 participants reported that they were currently receiving mental health treatment for mental health issues; none of them reported receiving treatment for addiction. Per AUDIT-C scores from the administrative records, patients reported a moderate level of drinking problems (mean 6.3, SD 2.4; out of a possible score of 12).

**Table 1:** Sample characteristics.

Variable		Patients (N=12)		Peers (N=11)	
**Gender, n (%)**					
	Male		11 (92)		9 (82)
	Female		1 (8)		2 (18)
**Race and ethnicity, n (%)**					
	African American/black		4 (33)		1 (9)
	White (non-Hispanic)		7 (58)		7 (64)
	Hispanic		0 (0)		3 (27)
	Other		3 (25)		0 (0)
Age, mean (SD)		58.9 (19.4)		48.3 (9.4)	
**Education (highest level), n (%)**					
	High school diploma/General Equivalency Degree		1 (8)		5 (46)
	Some college		2 (17)		3 (27)
	4-year college/university		5 (42)		2 (18)
	Graduate degree		4 (33)		1 (9)
**Smartphone comfort level (1-5 scale), mean (SD)**		4.5 (0.8)		4.4 (0.9)	
	1=very uncomfortable, n (%)		0 (0)		0 (0)
	5=very comfortable, n (%)		8 (67)		7 (64)
**Device used during the study, n (%)**					
	Personal device		8 (67)		5 (46)
	Study-provided iPod Touch		4 (33)		6 (55)

#### Peer Providers

A convenience sample of peer providers was recruited across 5 VHA facilities. Peer providers were eligible if they were veterans, worked with other veterans with alcohol use problems, and had been employed by VHA for at least 6 months. Approximately 71 peer providers were informed about the study through passive recruitment strategies, that is, mass emails to provider listservs and phone and in-person meetings with teams of peer support specialists and addiction therapists) rather than individual contacts. Overall, 13 peer providers contacted the study staff to express an interest and were enrolled, 2 of whom ultimately withdrew, yielding a sample of 11 (see sample characteristics in Table 1). Provider participants were mostly male (9/11, 82%), were white (non-Hispanic; 7/11, 64%), had at least some college education (6/11, 55%), and were 48.3 years old on average (SD 9.4 years). In addition, 3 participants were employed by the VHA as addiction therapists, and 8 were employed as peer support specialists. Among them, 5 participants reported working in their position for less than 3 years, and 6 participants reported working in their position for 3 years or more.

### Procedures

#### Method for Program Adaptation Through Community Engagement Steps

The M-PACE model guided adaptation of Step Away for the veteran population [4]. The first step involved creation of a steering committee with members who represented key stakeholders in adaptation of the intervention—that is, researchers, practitioners, program developers, and patients. Specifically, the committee for this study comprised 12 individuals: 6 research investigators (experts on addiction among veterans, evidence-based treatments for AUD, and mHealth), the app developer (PLD), 4 VHA administrators and clinicians in primary care–mental health integration and peer support services at both the local and national levels, and a representative of a Veteran and Family Engagement Council. The second step of the M-PACE model involved implementing the program as originally designed with patients and providers who will ultimately use the adapted intervention. This study’s target users were veterans who screened positive for hazardous drinking during primary care visits and peer providers who would provide telephone-based care to veterans using the app. The third step involved systematically soliciting feedback and suggestions for change from patients and providers, using both qualitative and quantitative research methods. Surveys with open-ended and scaled items were used to assess likes, dislikes, and recommendations for change during the course of the intervention and soon after its completion. The fourth step involved summarizing the quantitative and qualitative data and reviewing results with the steering committee. During the fifth step—adjudicating participants’ feedback—each suggestion for change was evaluated using 3 criteria: importance (degree to which the change will improve effectiveness of the intervention), feasibility (degree to which additional burden would be placed on patients, providers, or program developers), and congruence (degree to which the suggested changes work with or against the core change features of the intervention). After evaluating each suggestion, the steering committee decided whether to implement the suggested change.

#### Data Collection

After enrollment, patients and providers completed a baseline interview to assess demographics and rate their comfort using a smartphone on a 5-point scale (1=*very uncomfortable*, 5=*very comfortable*). Both patients and peer providers reported a high level of comfort in using these devices (see Table 1). Patients were also queried on their current receipt of mental health or addiction treatment, and peer providers were queried on their length of employment in the VHA. A research assistant (RA) then assisted participants with downloading the unmodified Step Away app to their iPhone (the app was only available on the iOS platform at the time) or a study-provided iPod Touch. The majority of patients used their personal device (8/12, 67%), whereas the majority of peer providers borrowed an iPod Touch (6/11, 55%). Participants were asked to use the app daily for 10 days, reviewing an average of 1 of the 10 modules per day, and to complete daily surveys (sent via email using REDCap) to provide feedback on each module’s usability. Participants were provided with a written guide to direct their review of the individual modules. This approach to data collection increases participants’ accurate recall of feelings and reactions to program material and obtains suggestions that are specific to elements of a program (eg, a given module) rather than generalized across program material [4]. The daily surveys consisted of 5 items involving quantitative ratings of the usefulness (1=*not at all useful*, 5=*very useful*) and difficulty of using a certain Step Away module (1=*not at all difficult*, 5=*very difficult*), and 3 open-ended questions asking what the participant liked, what they disliked, and what they would change about the module. With exception of the *Reminders* module, usability ratings for modules were completed by a majority of participants (see Table 2). Semistructured interviews were conducted by phone 2 weeks after enrollment to obtain global feedback from participants on the perceived utility of Step Away, facilitators and barriers to engagement with the app, and suggested modifications to enhance engagement and effectiveness with veteran primary care patients. App usage data were extracted from the app during the 2-week period to validate participants’ use of the app during their participation. Owing to limited Wi-Fi availability, 5 participants who borrowed an iPod Touch did not have their app usage data uploaded to the Cloud-based storage system where app usage was being tracked. Therefore, data on app usage were available for 18 participants (9 patients and 9 peers). These participants launched an average of 5.3 (SD=4.2) of the 10 app modules and reviewed the app for an average of 150.2 min (SD=43.3) over the 2-week period. Patient participants were paid US $25 for the follow-up interview, US $5 per Web-based survey, and an additional US $25 if they completed all 10 Web-based surveys. All participant procedures were approved by the local institutional review board.

**Table 2:** Usability ratings of the Step Away modules from veteran patients and peer providers.

Module	Total sample		Patients			Peers			Sample responses to free-text questions		
	Usefulness, mean (SD)	Difficulty, mean (SD)		Usefulness, mean (SD)	Difficulty, mean (SD)		Usefulness, mean (SD)	Difficulty, mean (SD)		Liked	Disliked/would change
Drinking patterns^a^	3.76 (0.9)	1.88 (1.1)		3.63 (0.9)	1.86 (1.2)		3.90 (0.7)	1.90 (0.9)		“Allows user to see how much they drink and the financial cost of drinking.” [Peer-004]	“Shorten, simplify. I find alcoholics are impatient while actively drinking.” [Peer-002]
Goals^b^	4.06 (0.9)	1.88 (0.8)		3.67 (1.1)	1.86 (0.9)		4.44 (0.7)	1.89 (0.8)		“Health and money as benefits of not drinking.” [Patient-001]	“Explanations were very long.” [Patient-005]
Rewards^c^	3.76 (1.3)	1.62 (1.2)		3.38 (1.1)	1.06 (0.2)		4.11 (1.4)	2.11 (1.5)		“Allows for custom input for rewards.” [Peer-012]	“Add reward choices that will appeal to lower income vets.” [Patient-008]
Cravings^d^	4.21 (1.1)	1.64 (1.2)		3.83 (1.3)	1.50 (0.8)		4.50 (0.8)	1.75 (1.4)		“You can isolate and identify specific triggers.” [Peer-013]	“A lot of reading and less interacting in this step.” [Peer-012]
Strategies^e^	4.00 (1.2)	1.93 (1.3)		4.00 (1.0)	1.57 (1.1)		4.00 (1.4)	2.29 (1.5)		“Good variety of strategies.” [Patient-003]	“Seemed aimed at heavy drinkers.” [Patient-008]
Support persons^f^	3.81 (1.0)	1.41 (0.7)		3.63 (1.1)	1.56 (0.9)		4.00 (1.1)	1.25 (0.5)		“Actually putting in the number and email of the person.” [Peer-001]	“Prompt user to check in with someone every day.” [Peer-006]
Reminders^g^	3.60 (1.1)	1.60 (1.1)		3.29 (1.1)	1.71 (1.3)		4.33 (0.6)	1.33 (0.6)		“Different ways to personalize reasons for change (eg, photos of loved ones).” [Patient-003]	“More text space to provide reasons for change.” [Patient-010]
High risk^h^	3.75 (0.9)	1.33 (0.8)		3.67 (0.8)	1.00 (0.0)		3.83 (0.9)	1.67 (1.0)		“Allows me to see what time I am more at risk to drink.” [Peer-009]	“Provide link to supports as way to deal with high-risk times.” [Patient-013]
Moods^i^	3.94 (0.7)	1.34 (0.6)		3.86 (0.7)	1.14 (0.4)		4.00 (0.7)	1.50 (0.7)		“Keeps weekly track of fluctuations in mood.” [Patient-011]	“More information regarding graphs.” [Patient-010]
New activities^j^	4.23 (0.8)	1.54 (0.9)		4.40 (0.9)	1.40 (0.9)		4.13 (0.8)	1.63 (0.9)		“Easy to customize. Allows you to plan ahead for a high-risk time.” [Peer-013]	“Expand to track activities taken up instead of drinking.” [Patient-010]

^a^N_Total sample_=21, N_Patients_=11, N_Peers_=10.

^b^N_Total sample_=18, N_Patients_=9, N_Peers_=9.

^c^N_Total sample_=17, N_Patients_=8, N_Peers_=9.

^d^N_Total sample_=14, N_Patients_=6, N_Peers_=8.

^e^N_Total sample_=15, N_Patients_=8, N_Peers_=7.

^f^N_Total sample_=16, N_Patients_=8, N_Peers_=8.

^g^N_Total sample_=10, N_Patients_=7, N_Peers_=3.

^h^N_Total sample_=12, N_Patients_=6, N_Peers_=6.

^i^N_Total sample_=16, N_Patients_=7, N_Peers_=9.

^j^N_Total sample_=13, N_Patients_=5, N_Peers_=8.

#### Data Analysis

Descriptive statistics were calculated for quantitative items from the daily surveys and the follow-up interview. Textual data from the open-ended questions of the daily surveys and the follow-up interviews were analyzed using techniques for rapid qualitative analysis recommended by Hamilton [26]. Specifically, 2 RAs reviewed responses to the daily surveys, listened to the audio recordings from the semistructured interviews, and took detailed notes using a template to summarize responses to the survey and interview questions and document preliminary themes related to facilitators and barriers to engagement and suggested modifications. The RAs then copied these notes into an Excel matrix to compare the preliminary themes for each question (columns) across participants (rows). This matrix was organized such that the summary of participants’ response to a question and the preliminary theme were entered into each cell. Furthermore, 2 study investigators independently reviewed the composite matrix of these summarized responses to identify global themes in the data corresponding to domains of facilitators and barriers to engagement and suggested modifications to enhance the engagement and effectiveness of Step Away among veterans who engaged in hazardous drinking. The investigators then met to review their independently derived lists of themes and engaged in a consensus process to rectify disagreements and finalize the themes in each of these domains.

## Results

### Usability Ratings of the Step Away Modules

Table 2 provides the daily survey ratings of each Step Away module in terms of usefulness and difficulty. In general, the modules were rated as moderate-to-high in usefulness and low in difficulty. Table 2 also provides sample responses to the free-text questions regarding what participants liked, what they disliked, and what they would change about each module. The suggestions for change typically followed directly from what participants reported disliking about a module; therefore, responses to those questions were combined for summarizing and reporting. Across modules, participants reported liking the ability to customize and personalize information as well as the ability of different modules to track changes in the information that users provided. Aspects of the modules that participants tended to dislike or would change pertained to the heavy amount of reading, a desire for the module to interact more with the user, and expanding the extent to which modules could track the information entered by the user. Notably, across modules, responses to the free-text questions addressed themes of money and finances (eg, liking how the app highlighted the financial costs of drinking and suggestions to add options for recreational activities for low-income veterans).

### Perceived Utility of Step Away

When asked, “Will the app help veterans reduce the amount they drink or how often they drink?,” 22 of the 23 participants answered affirmatively. When asked, “How likely are you to continue using Step Away?,” 15 of the 23 participants indicated plans to continue using the app or said they would continue to use it if it were available on their personal phone. Patient participants noted the benefits for increasing their access to care and ability to monitor their drinking over time:

It would help. Not a lot of people are in an area where there’s availability for help. It’s helped me already.

Patient-009

It's good because you can put in the exact number of drinks and track your progress. I think I personally drank less while using it.

Patient-011

Peer participants also noted the benefits of the app for reducing a patients’ drinking, although they added that this would depend on another person keeping the patient accountable for using it:

If they have a solid support system that reminds them of using the app it would be helpful.

Peer-002

Yes, it will hold them accountable and help them. As long as they’re reminded to use it, it would help.

Peer-010

### Facilitators and Barriers to Engagement With Step Away

Regarding facilitators to their engagement with Step Away, participants noted the (1) reminders on high-risk times and situations; (2) encouraging rewards for reaching one’s drinking goals; (3) personalized feedback, particularly around the financial costs and health consequences of alcohol use; (4) ability to customize one’s drinking goal and other features of the app; and (5) measurement capabilities, such as the app’s ability to track one’s drinking and progress toward their drinking goal (see Table 3). In terms of barriers to engagement with the app, participants noted (1) key features of Step Away that felt hidden or insufficiently highlighted (eg, the *Get Help* feature and options for customization), (2) concerns that some aspects of the interface could limit user engagement (eg, not user-friendly for elderly patients because of the small font size, not enough visuals and graphics and the app is *text heavy*), and (3) privacy concerns that were insufficiently acknowledged.

**Table 3:** Facilitators and barriers to engagement with Step Away.

Themes		Sample quotations
**Facilitator**		
	Reminders on high-risk times and situations	“[I like that it] provides education and awareness of risky situations and times of drinking.” [Patient-005]; “I like that it reminded me when I was coming up on a time I was going to drink.” [Peer-006]
	Encouraging rewards for reaching drinking goals	“Rewards for milestones reached were helpful. Sometimes veterans don’t know what to do for themselves as a reward instead of drinking. Typically, they would go out and have a drink.” [Peer-001]
	Personalized feedback regarding financial costs and health consequences of alcohol use	“Information about amount of money spent on alcohol was useful” [Peer-004]; “Good information in terms of caloric impact of my drinking and how alcohol may be affecting my problems with weight control.” [Patient-013]
	Ability to customize one’s drinking goal and various app features	“It allowed me to set up a schedule to be notified; it engaged me and helped me be involved…it made it more personalized.” [Patient-008]; “I like that it gives a choice of moderation. That makes it helpful for people who might not want to fully quit but still want help.” [Peer-011]
	Measurement capabilities (eg, ability of app to track drinking and progress toward goals)	“If you want to change something, measure it. [The app] helps vets pay attention to what they’re doing and keep track of progress so they have an idea of what they need to change.” [Patient-010)
**Barrier**		
	Key features appeared hidden or were insufficiently highlighted: The “Get Help” feature; more options for customization	“I tend not to click on help icons. One reason is because I typically don’t find the answer I’m looking for and another is because I did not feel the need to use the feature.” [Patient-010]
	Concerns that some aspects of the interface could limit user engagement: Not user-friendly for elderly patients (eg, small font size); “Text heavy”—not enough visuals and graphics.	“It was hard to read. It would be nice if they could be magnified for people with older eyes.” [Patient-013]; “I don’t think it would be helpful for vets who are actively drinking. Too much reading.” [Peer-004]
	Privacy concerns insufficiently acknowledged	“Trust issues with government and VA. Fear of breach of confidentiality. Those who are new to sobriety might be overwhelmed.” [Peer-002]; “Provide a rationale as to why we are putting contacts in, for example, including that [providing personal information] typically helps people for reasons x, y, and z; otherwise it could be triggering.” [Patient-005]

### Suggested Modifications to Enhance Engagement and Effectiveness of Step Away Among Veterans

The qualitative interviews yielded themes to enhance engagement and effectiveness of Step Away with the veteran population (see Multimedia Appendix 1, Column 1 for a bulleted outline of the suggested modifications). One theme focused on modifying the appearance and design of the app to include more veteran-centric content. Another theme related to the need to revise the text to increase the usability and relevance of the app for older and/or lower-income veterans. Other veteran-centric modifications included adding links to resources and services for veterans in crisis or seeking treatment; adding information on drinking problems among veterans; adding more preprogrammed response options relevant to veteran preferences and needs; and addressing potential privacy concerns among veterans such as whether the app data would be entered into patients’ Veterans Affairs (VA) medical records. Although many suggestions focused on how to repurpose the app for veterans *per se*, other modification themes focused on ways to increase the usability of the app more generally. For example, participants suggested more orientation to the app when users set up the program; more use of graphics to track progress toward one’s drinking goal over time; more interactive features, and increasing access and engagement with the app content more generally.

### Adjudicating the Suggested Modifications to Step Away

Following the analyses, 2 meetings with the steering committee were convened. The first meeting (daylong) was held in person, with remote members attending by phone. Committee members downloaded Step Away to their iPhones or a study-provided iPod Touch to review the app before the meeting. During the meeting, a demonstration of Step Away’s content and functionality was given by the lead author. Participant feedback and other summary findings were then reviewed, and the suggested modifications to Step Away were discussed and operationalized. Committee members were asked to evaluate the suggested modifications according to 3 criteria: *importance*—the degree to which the change could enhance Step Away’s effectiveness and engagement with the veteran primary care population; *feasibility*—how burdensome the change would be to veterans, peer providers, and/or app developers; and *congruence*—did the change work with or against the core components of Step Away.

Following this meeting, a list of proposed changes to Step Away to create a veteran version of the app was drafted by the lead (DMB) and senior authors (PLD) and emailed to committee members. Members rated each of the proposed changes in terms of whether it was (1) *essential*, (2) *nice to have, but not essential*, or (3) *not essential* to incorporate into the veteran version of the app. Proposed changes that received a rating of *essential* by a majority of the committee members and were retained in the final list are shown in Multimedia Appendix 1. Changes that were not viewed as *essential* by a majority of committee members included many of the interactive features that were suggested by participants—for example, voiceover audio, educational games, and link to an online forum. These changes were viewed by the committee as too costly and resource intensive to incorporate into the app’s format and were therefore not included into the final list of changes.

### Creating the Veteran Version of Step Away (Stand Down: Think Before You Drink)

The lead and senior authors had a series of meetings to review the progress on the planned changes, discuss and edit wireframes of the changes, and conduct beta testing of the initial versions of the revised app. To solicit feedback on specific content that was being considered for inclusion in the app, a meeting was convened with members of the Veteran and Family Engagement Council at the local facility. This council assists with the development and implementation of projects and initiatives at the facility to ensure that the perspectives of veterans and their family members are incorporated. After all the changes to Step Away were completed and beta tested by the lead author and an RA, a second meeting of the steering committee was convened by phone. Before this meeting, an initial version of the repurposed app—*Stand Down: Think Before You Drink* (or *Stand Down*)—was made available to members for review. In the military, *Stand Down* refers to a period of recovery after a state of high alert; and among veterans, the term also refers to multiday events in providing them with resources, services, and referrals to additional services. During the meeting, major changes to the app were reviewed and feedback was solicited from the committee members regarding additional changes to be made to the app. On the basis of this feedback, minor changes were made to Stand Down, and the app was launched for research use in the iTunes store [27].

## Discussion

### Principal Findings

The goal of this study was to use the M-PACE model to repurpose the Step Away mobile intervention system for US veterans. In particular, we sought to create a version of the app that would enhance engagement and effectiveness with veterans in primary care who screened positive for hazardous drinking, many of whom are in mid-to-late life [21,22]. A key advantage of using the M-PACE model was the ability to obtain feedback on both the individual modules of Step Away and global feedback on the app. Feedback on both facilitators and barriers to engagement with Step Away was critical to this process, which helped the Steering Committee adjudicate which modifications to this app were most essential and how best to implement them.

One strength of the app from the perspective of participants was the personalized feedback provided via the *Drinking* *Patterns* module. Personalized, norm-based feedback is viewed as an essential component of brief interventions for drinking problems [12] and is a feature not currently incorporated into other mobile apps for drinking problems for veterans [28]. Feedback on the financial costs and health consequences of drinking were highlighted by participants and the steering committee as common concerns of the target population that should be emphasized in the veteran version of the app. These issues are now highlighted to a greater extent in Stand Down than in Step Away. Another perceived strength of the app was the ability of users to customize the content to fit their needs and preferences, such as their drinking goal, rewards, nondrinking activities, and reminders. Such a personalized approach to care is consistent with a broader shift in addiction and mental health treatment toward more recovery-oriented and patient-centered approaches to care designed around patients’ unique goals and priorities for care [29,30]. To this end, Stand Down includes more preprogrammed recreational activities for veterans including a list of organizations that support veteran activities and events. The measurement-based care capabilities of Step Away in terms of tracking users’ alcohol use over time and progress toward their drinking goal was also viewed as a strength by participants. Consistent with a broader effort in VHA and the addiction treatment literature of measurement-based care [31], this feature of Step Away was expanded in Stand Down by showing users’ weekly progress toward their drinking goal on the home screen.

One potential barrier to engagement with Step Away was both the size and the amount of text throughout the app’s modules. Small font size may be particularly salient for the target population, given that many veterans treated in primary care in VHA are mid-to-late life and may have visual impairments [32]. Consistent with this, prior research has recommended using larger font on mHealth tools targeted toward older adults [3]. Relatedly, other research has suggested minimizing text in mHealth tools when possible to mitigate the potential for fatigue [33]. Accordingly, a key modification to create the Stand Down app involved a careful review of the app’s text files to identify opportunities to shorten descriptions, break up long paragraphs, and enumerate key information. These changes, along with more proactive presentation of app content via the Message of the Day, were intended to enhance the overall usability of the app for the target population.

In addition to modifying Step Away to address the characteristics of the veteran population and increase its usability, other changes involved alterations to the *look and feel* of the app to incorporate more veteran-centric content. For example, we replaced both the app icon and the splash page that users would see when they launch the app with an image of an American flag (see Multimedia Appendix 1), embedded videos of veterans describing their recovery from drinking problems, and rebranded the app name. These changes are unlikely to have an adverse impact on the effectiveness of the Step Away program and may have the benefit of increasing the extent to which veterans identify with the app. This approach to tailoring an mHealth tool for the veteran population was employed successfully in a recent study that modified a Web-based intervention for anxiety and depression for rural veterans [20]. This study extends this principle of adapting mHealth tools for veterans to mobile apps and to the treatment of hazardous drinking in this population.

### Strengths, Limitations, and Future Directions

The design of this study included a number of strengths, many of which are inherent to the M-PACE model. Specifically, we used a mixed-methods approach to data collection to obtain comprehensive feedback from key consumers (veteran patients and peer providers) on the unmodified app. Drawing on the principles of community-based participatory research [34], a diverse set of stakeholders with expertise in addiction, evidence-based treatments for AUD, primary care–mental health integration, and mHealth was used to adjudicate the suggested changes to Step Away. Furthermore, the iterative and systematic approach to adjudication helped to identify which changes to the app can maximize its effectiveness with veterans while also being feasible to implement and congruent with the core components of Step Sway [4]. These benefits of the M-PACE model notwithstanding, there may be opportunities to extend the model by incorporating elements from the field of instructional design [35]. For example, cycles of formative evaluation are common in instructional design models as they allow for revisions of mHealth tools before implementation. Such an approach could be incorporated into the M-PACE model after the adjudication process by soliciting feedback on the new tool with the same consumers who initially reviewed the unmodified tool. This would permit assessment of whether the suggested modifications were incorporated sufficiently or whether further revisions to the mHealth tool are needed prior to its implementation with the target population.

Among the study limitations, the patient feedback was based on a sample from a single VA Health Care System, which may not generalize to the perceptions of veterans in other clinics and geographical regions. This limitation was somewhat countered by soliciting feedback from peer providers across multiple geographical regions in the United States. Furthermore, a minority of both the patient and peer providers who were contacted about the study agreed to participate; for patients, this may reflect the fact that many veterans who screen positive for alcohol use problems are not interested in receiving help. Consequently, feedback from these highly self-selected groups may not be representative of the larger samples of veterans and providers who could conceivably use the app. A larger sample size of both patients and peer providers may also have been advantageous. However, a substantial portion of the participant feedback involved qualitative data, and sample sizes of 10 or more are often sufficient for reaching thematic saturation and conducting group comparisons in qualitative research [36,37]. The modest launch rate (5.3 out of the 10 app modules) and the lower number of daily surveys completed for some of the Step Away modules (eg, *Reminders*) also suggests that participants may not have reviewed the full menu of tools available in Step Away. Potentially, this modest level of app usage may be improved by the modifications that were made through the M-PACE process. Finally, this study did not provide data on the acceptability or utility of the Stand Down app. Pilot testing of this veteran-focused app is a critical next step in this program research. Should it prove effective in future research for improving the drinking outcomes of veterans engaging in hazardous drinking, subsequent implementation research should focus on how best to enhance the reach of the app with the target population. Our involvement of key stakeholders at the national level of VHA in this early stage of this program of research should ultimately facilitate such efforts.

### Conclusions

We used the M-PACE model to repurpose the Step Away mobile intervention system to target the characteristics, needs, and preferences of veterans who are identified in primary care settings as engaging in hazardous drinking. We envision the approach outlined here as a generalizable method that other researchers can follow to systematically tailor an mHealth tool to maximize engagement and effectiveness of an app with a patient population for which the app was not originally designed. Ultimately, the use of Stand Down may serve as an innovative, low-cost means of overcoming barriers to access and engagement in alcohol use treatment among veteran primary care patients. Our preliminary study of this app would benefit from a follow-up study in a larger population and involving patients across multiple VA medical centers.

## Appendix

Multimedia Appendix 1 Modifications to Step Away to create a veteran version of the app (“Stand Down: Think Before You Drink”).

## Abbreviations

AUD: alcohol use disorder
AUDIT-C: Alcohol Use Disorder Identification Test for Consumption
iOS: iPhone Operating System
M-PACE: Method for Program Adaptation through Community Engagement
mHealth: mobile health
RA: research assistant
VA: Veterans Affairs
VHA: Veterans Health Administration
