# Supplementary material for: From “Step Away” to “Stand Down”: Tailoring a Smartphone App for Self-Management of Hazardous Drinking for Veterans
Source: JMIR Mhealth Uhealth. 2020 Feb 13;8(2):e16062. doi: 10.2196/16062 (PMC7055774; doi:10.2196/16062)
Supplement: Multimedia Appendix 1 [file mhealth_v8i2e16062_app1.docx]

*Multimedia Appendix 1. Modifications to Step Away to create a veteran version of the app (“Stand Down: Think Before You Drink”)*

| ***Suggested modifications*** | ***Specific changes approved by the  Steering Committee*** | ***Sample screenshots*** |
| --- | --- | --- |
| 1. Modify the appearance and design to include more veteran-centric content:    - Add images relevant to military/veteran culture.    - Include testimonials from other veterans. | - - Re-brand the app with a name, icon, and image that connotes military/veteran culture.   - Adding a “Veterans’ Stories” module with links to videos from <https://maketheconnection.net> of veterans talking about their recovery from alcohol use problems. | 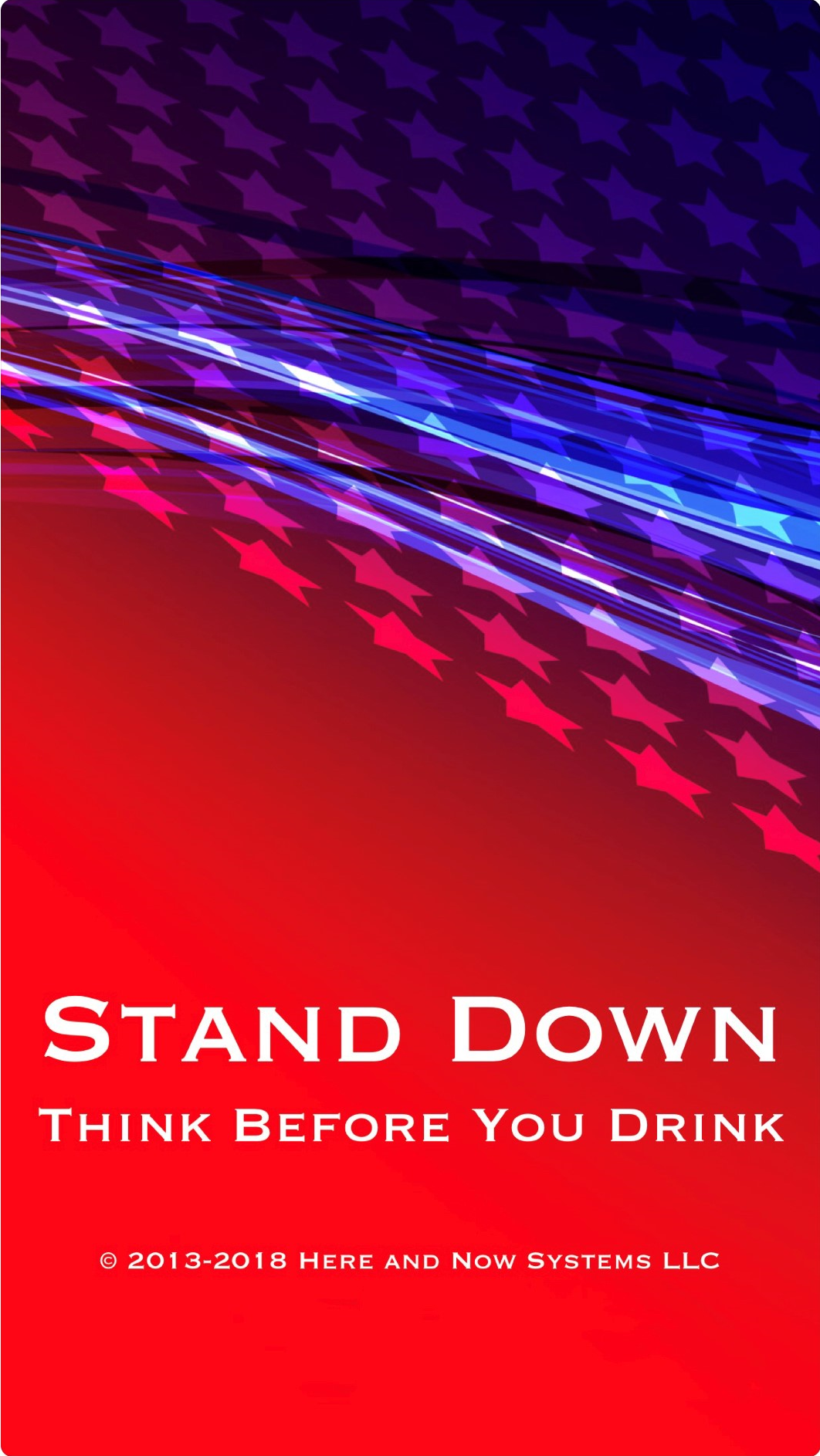 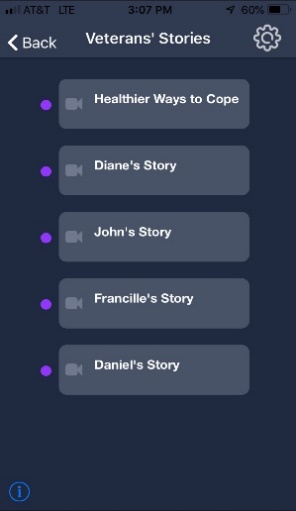 |
| 1. Revise text to increase usability and relevance for target population (e.g., older veterans, lower-income veterans). | - - Increase the font size throughout the app.   - Edit the text files to reduce the word length and include more references to veterans and their concerns (e.g., health problems, finances). | 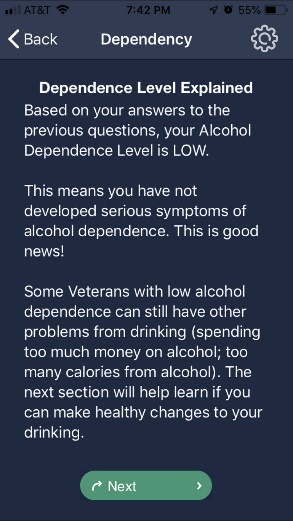 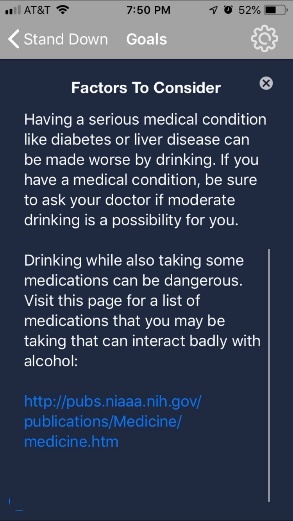 |
| 1. Add links to resources and services for veterans in crisis or seeking treatment. | - - In the ‘Get Help’ feature, add options for calling the Veterans Crisis Line, finding a VA treatment program or Vet Center, or calling the AA national hotline. | 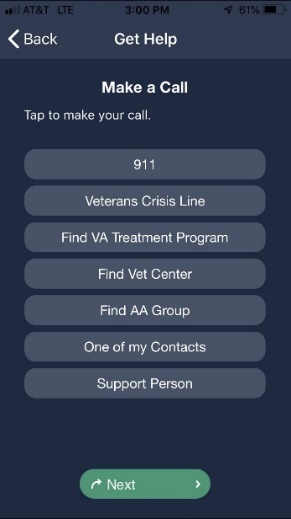 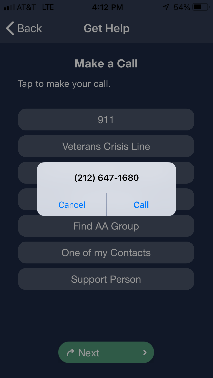 |
| 1. Add information on veterans and alcohol use problems (e.g., risk factors) | - - In the ‘Cravings’ module, adding text on anger and frustration and thoughts of past trauma as common triggers for drinking among veterans. | 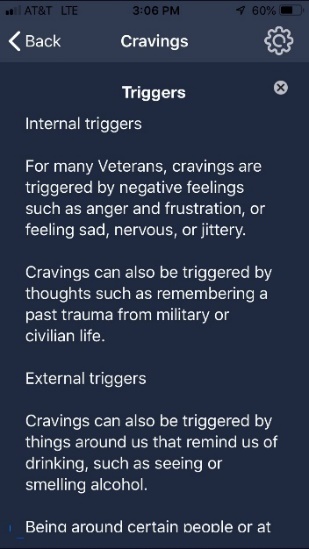 |
| 1. Adding more pre-programmed response options relevant to veteran preferences and needs. | - In the ‘New Activities’ module, populate each category with more options for low-income veterans. - Adding a “Veterans Events and Services” category to the ‘New Activities’ module and providing a list of veteran organizations. | 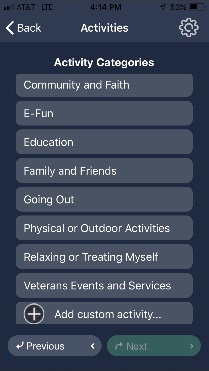 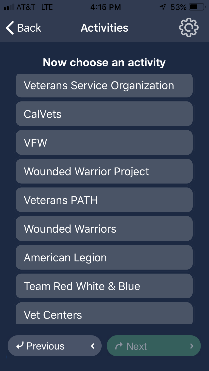 |
| 1. More orientation to the app when users set up the program (e.g., who the app is for, key features, and options for customization). | - Adding information during the set-up process that specifies that the app is for veterans and a tab describing “How it Works.” | 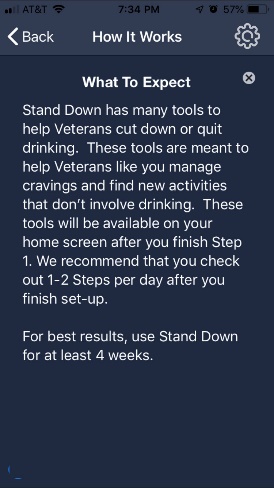 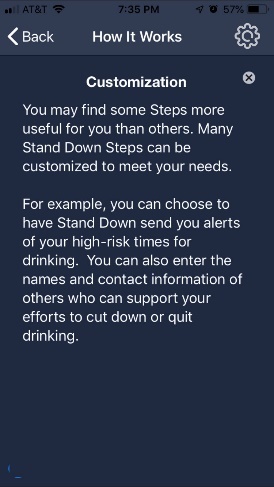 |
| 1. Addressing potential privacy concerns among veterans. | - In the Welcome Screen, stating that the app was made with input from other veterans and the information provided is not recorded in veterans’ VA medical records. | 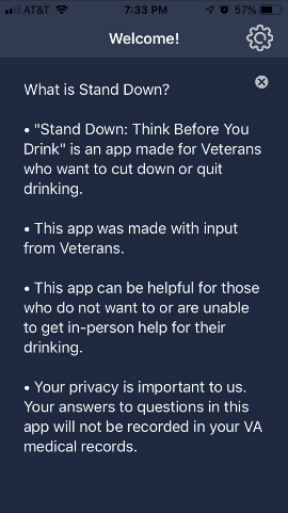 |
| 1. Graphics to track progress towards drinking goals, and more interactive features (e.g., voice-over audio, educational games, links to a Twitter-style messaging forum for other app users). | - - Revise the Home Screen of the app to show the user’s drinking goal and their progress towards this goal. | 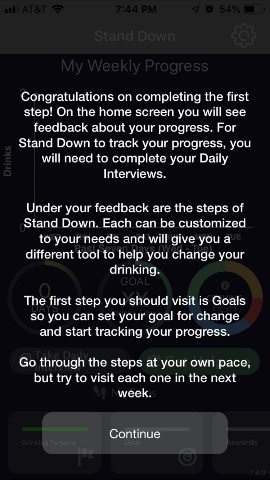 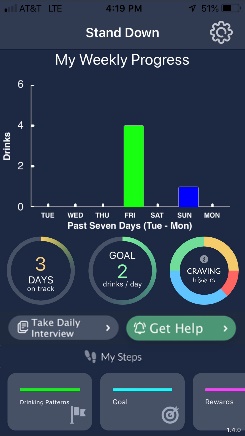 |
| 1. Increasing access and engagement with the app content more generally (e.g., motivational statements, pop-ups, notifications). | - A “Message of the Day” pop-up that includes a motivational statement or suggestion on a module to review. | 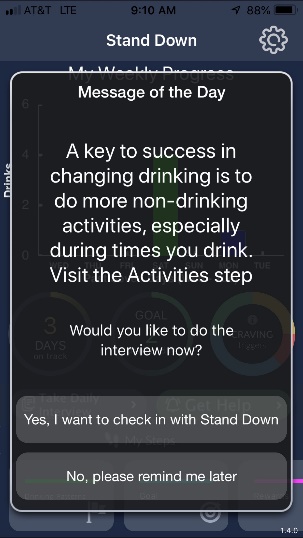 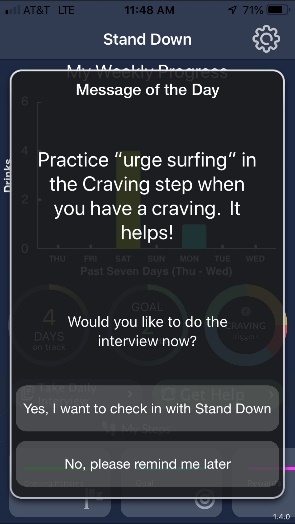 |
